# Managing Newborn Screening Repeat Collections for Sick and Preterm Neonates

**DOI:** 10.3390/ijns10030063

**Published:** 2024-09-16

**Authors:** Ronda F. Greaves, Jo-Ann Northfield, Lauren Cross, Nazha Mawad, Thanh Nguyen, Maggie Tan, Michele A. O’Connell, James Pitt

**Affiliations:** 1 Victorian Clinical Genetics Services, Murdoch Children’s Research Institute, Parkville, VIC 3052, Australianazha.mawad@vcgs.org.au (N.M.); thanh.nguyen@vcgs.org.au (T.N.); maggie.khon@vcgs.org.au (M.T.); james.pitt@vcgs.org.au (J.P.); 2 Department of Paediatrics, University of Melbourne, Parkville, VIC 3052, Australia; michele.oconnell@rch.org.au; 3 Department of Neonatal Medicine, The Royal Children’s Hospital, Parkville, VIC 3052, Australia; lauren.cross@rch.org.au; 4 Department of Endocrinology and Diabetes, The Royal Children’s Hospital, Parkville, VIC 3052, Australia

**Keywords:** prematurity, low birth weight, transfusion, sick neonate, quality indicators, congenital hypothyroidism, transient hypothyroidism, congenital adrenal hyperplasia

## Abstract

Some preterm and sick neonates have altered biochemical profiles and follow-up newborn screening (NBS) collections are recommended. The Victorian NBS program historically recommended repeat collections for babies with birth weight < 1500 g (managed by the maternity service provider) and 3 weeks post-transfusion (managed by the laboratory). We aimed to determine adherence to current guidelines and review the guidelines to improve NBS performance. To do this, we audited data from 348,584 babies between January 2018 and June 2022. Babies with a recorded birth weight of <1500 g were filtered for inclusion. For the overall review and visualization of the protocol, we sourced information from the literature, our professional society and tertiary hospital services. A total of 2647 babies had a birth weight recorded between 200 and 1499 g. Of these, 2036 (77%) had a second sample collected, indicating that >1 in 5 babies were not receiving a follow-up collection. Our timing of repeat collections for transfused babies, requiring a 3-week follow-up collection, was longer than in other Australasian jurisdictions. A new combined “sick–prem protocol” was launched to support repeat collections and after a 1-year review achieved 95% compliance. We recommend NBS laboratories audit preterm and sick neonate repeat collections to ensure appropriate follow-up. This should be supported with a visual process map to aid education and compliance.

## 1. Introduction

Low birth weight (LBW), preterm and sick neonates are a small but complicated cohort for newborn screening (NBS) programs. They require additional consideration and protocols to ensure they are appropriately screened [1,2]. The management of this cohort in our jurisdiction (Victoria, Australia) has traditionally been by the maternity service providers for the LBW babies following our repeat collection guideline, and for sick babies requiring a blood (red blood cell) transfusion this was managed by the NBS laboratory team. Some babies are both sick and LBW and this is complicated for the health care providers in the ward and the laboratory. Clear guidance to facilitate repeat collections is therefore needed. However, for this special cohort of neonates, there are gaps in evidence-based best practices to inform the re-sampling requirements.

Neonatology is a young discipline that has developed in parallel over the same period as NBS. Today neonatologists are very good at keeping preterm (20 to <37 weeks gestation) babies alive. This cohort now makes up about 8.2% of births in Australia and globally ranges from 4 to 16% of all births. A subset of these includes very and extremely preterm or LBW births [3,4]. This improvement in care coincides with the increased number of preterm babies needing follow-up by NBS programs [3,5]. LBW (<2500 g) can be caused by intrauterine growth restriction, prematurity or both, and therefore because LBW considerably overlaps with preterm birth, and its measurement is generally considered more reliable than gestational age (GA), it has been used as the primary decision point for all preterm repeat collections in Australasia [6,7]. We note, though, that being born too small is conceptually different from being born too early. Irrespective, it is the very preterm (28 to < 32 weeks), extremely preterm (<28 weeks) and very LBW neonates that provide the significant challenges for improving morbidity and interpreting laboratory test results. In addition, the sick neonate who requires a transfusion of blood products further challenges NBS programs to ensure false negative screening results do not occur.

Certainly, the smaller the baby, the more immature the organ systems are, and in the case of the endocrine systems this can result in false-negative NBS results for thyroid screening. Thyroid hormone levels are known to decrease in the first week of life and probably reflect a transient depletion of thyroid hormone reserves. This nadir is more pronounced in preterm neonates, especially in infants <30 weeks gestation [8]. This also coincides with an immature hypothalamic–pituitary axis where the ability to produce TSH in response to low T4 and T3 is blunted [9]. As such, the first measurement of TSH in the usual timeframe of 48–72 h of life may miss cases of congenital hypothyroidism (i.e., false negative); and conversely, if thyroxine is the first tier measurement, false positive cases may occur [10]. Likewise, preterm babies have a persistent fetal adrenal zone and therefore some also require a follow-up collection when screening for congenital adrenal hyperplasia (CAH) [11,12]. Hence the need and routine recommendation for repeat collection(s) in these instances.

With the introduction of an increased number of conditions into NBS programs globally, the complexities of managing the preterm and sick neonates across all conditions are challenging. Recently we introduced CAH screening, with other conditions planned for introduction in quick succession [13]. With more conditions come some compromises in timing of repeat collections to avoid excess sample collections and iatrogenic anemia. Given the complexities involved, we had previously received requests from our maternity service providers for a visualization of the repeat collection cascade for preterm and sick neonates. Hence, it was timely to review (audit) our processes and ideally set up a system to manage repeat collections for preterm and sick neonates that was robust, evidence-based and harmonized with other Australasian programs.

We therefore aimed to (a) determine adherence to our current guidelines; and (b) review and revise (if required) the guidelines with visualization to improve NBS performance.

## 2. Materials and Methods

### 2.1. Victorian NBS Process at Time of Audit Initiation

The Victorian Newborn Screening program services approximately 80,000 babies born annually. On arrival at the laboratory, one 3.2 mm spot is punched from the card for each first-tier assay. Commercial kits are run for TSH, 17 hydroxy progesterone (17OHP) and immunoreactive trypsin (IRT) on the Genetic Screening Processor (GSP^®^) from Revvity (Turku, Finland). The first-tier metabolic screening of amino acids and acyl carnitines is run by an in-house method on Waters Xevo mass spectrometry instruments. Spinal muscular atrophy (SMA) and severe combined immunodeficiency (SCID) by a hyper-automated PCR procedure were being prepared for introduction in 2023. Other conditions planned for implementation are galactosemia, X-linked adrenoleukodystrophy and sickle cell disease screening in 2024, 2025 and 2026, respectively.

At the time of the audit (in 2022), the Victorian NBS program collection guideline recommended routine collections for:

- All babies—between 48 and 72 h of life. From 2020, i.e., the start of the COVID-19 pandemic, samples were accepted down to 36 h of age without the need for a repeat collection.
- Low birthweight babies—babies with a birth weight < 1500 g required a regular collection and then a follow-up collection at 2 weeks (1000–<1500 g at birth) or 3 weeks (<1000 g at birth) of age. Organization of the repeat samples was managed independently by the maternity service provider without prompting from the NBS laboratory. The NBS laboratory was only involved as needed to prompt follow-up of borderline or screen positive results. Therefore, if the repeat collection did not happen because it was missed by the maternity service provider, the NBS lab did not have a role in recognizing this.
- Transfused neonates—babies who received any form of transfusion prior to the routine NBS collection, required an early sample (pre-transfusion), a 48 h post-transfusion, plus a 3-weeks post-transfusion. These repeat collections were managed by the NBS laboratory; i.e., where electronic letters were sent out to remind providers to perform the repeat collection and follow-up of outstanding samples.

### 2.2. Audit of Repeat Collection Guideline for Preterm Neonates

Babies with first samples received between January 2018 and June 2022 were included in the review. Babies with a recorded birth weight of <1500 g were filtered for inclusion as the denominator and the number of these babies that had more than one dried blood spot (DBS) collection was used as the numerator to give a percentage of preterm babies with a follow-up collection. As this was a quality improvement audit and part of the routine continual improvement management practices of the NBS program, ethical approval was not required.

### 2.3. Review of Evidence and Harmonization

For the overall review and visualization of the protocol, we sourced information from the literature and professional bodies.

- The USA’s Clinical Laboratory Standards Institute (CLSI) document NBS03 was extensively reviewed as part of the literature and review looking for their recommendations and the evidence to support this [1].
- The Human Genetics Society of Australasia NBS Committee was the professional body consulted, and each of the five other jurisdictions was asked (1) what their current recommendations were for preterm and transfused neonates, and (2) what is the evidence for their recommendation [14]?
- The World Health Organization definitions for low birthweight [15] and prematurity [16] were used to compare with other recommendations—Table 1.

### 2.4. New Protocol Design

The initial protocol design was performed in Microsoft PowerPoint, with changes recorded as new individual slides with dates and reason for change noted. Input was also invited from Victorian tertiary hospital services that had special care nurseries.

### 2.5. One Year On—Review of Impact of New Protocol

One year of data (May 2023 to April 2024) was retrieved from our laboratory information management system to review whether the change to the preterm protocol had resulted in improved second sample collection. Qualitative feedback was sought from the laboratory team on the effect of this change on their work. Anecdotal feedback was sought from the maternity service providers via our NBS Educator and review of our management system for instances of non-compliance.

### 2.6. Statistical Analysis

The patient data were exported from our laboratory information system into a CSV file in Microsoft Excel. Data were sorted based on birth weight (and gestational age for the updated SP protocol only) for inclusion and a simple count was performed to show if these babies had received at least a second collection. Descriptive calculations were performed in Microsoft Excel and Stata-18.0. Q-Pulse (Ideagen, Nottinghamshire, UK) was used to manage the audit.

## 3. Results

### 3.1. Audit of Current Repeat Collection Guideline for Preterm Neonates

A total of 348,584 NBS babies were screened between January 2018 and June 2022. Of these, 2647 babies had a birth weight recorded between 200 and 1499 g. From this subset, 2036 (77%) had a second sample collected, indicating that >1 in 5 babies were not receiving a follow-up collection.

### 3.2. Review of Evidence and Harmonization

The CLSI document was extensively reviewed, and this provided some recommendations for repeat collections. This included that transfusions were specifically whole blood transfusions and that extra corporeal membrane oxygenation (ECMO) babies should be included [1]. Representative(s) from all six Australasian jurisdictions were present at the HGSA Newborn Screening Committee meeting and responded to the questions that formed the information provided regarding the transfusion protocol. The information on LBW repeat collections was previously collated as part of a publication on harmonization of congenital hypothyroidism screening [7] (Table 2).

The consensus was that the evidence for the collection time frame for transfusions was not clear. Our Victorian time of repeat collections for transfused babies, requiring a 3-week follow-up collection, was longer than that in other Australasian jurisdictions, i.e., ours was at 3 weeks whereas others were either 1 or 2 weeks post-transfusion.

### 3.3. New Sick–Prem (SP) Protocol

**Figure 1:**
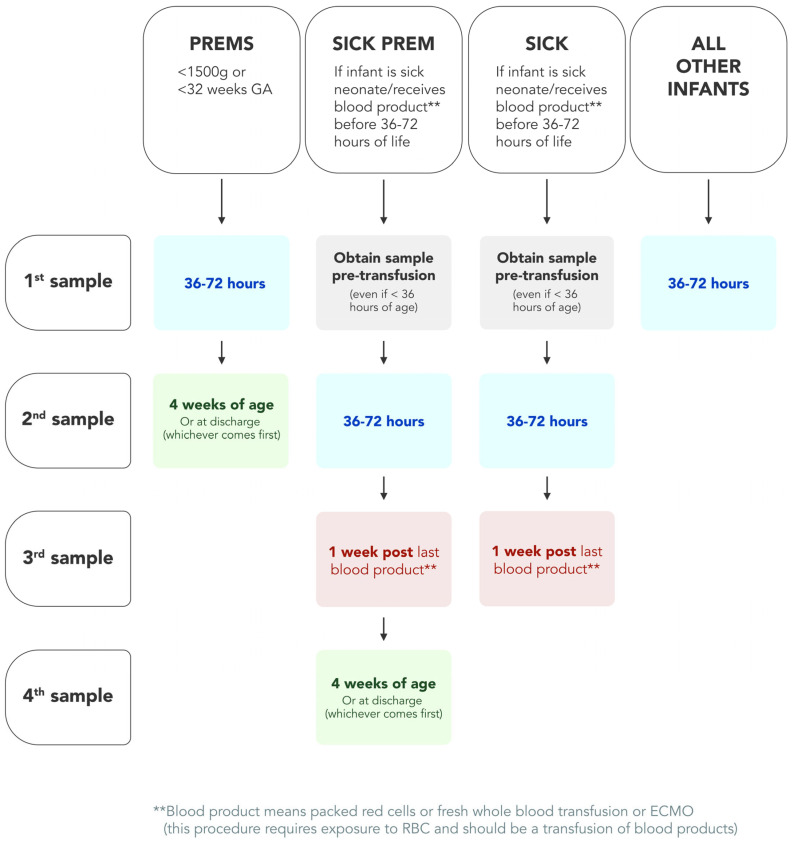
Revised newborn screening visual process map for sick and preterm neonates in Victoria Australia; effective from Monday 17 April 2023. Follow-up collections occur outside of this protocol for NBS results deemed screen-positive, i.e., requiring a re-collection or referral to a clinical team.

A new combined “sick–prem protocol” was launched (17 April 2023) to support repeat collections (see Figure 1). Under this protocol, all sick and preterm neonatal repeat collections are now managed by the NBS laboratory. At the time of data entry, any sample that meets the criteria has an “SP” flag added to the sample. For transfusions, the SP flag is added, and we also add the specific transfusion flag “Tx”. This initiates an electronic repeat collection letter to be generated and sent from the laboratory information management system. Follow-up reminder letters are also scheduled using this electronic process. If a repeat collection sample is not received, our NBS nurse educator phones the relevant maternity service provider to follow up. For the preterm neonates, the NBS laboratory closes the repeat collection request (for babies with screen negative results) once a sample has been received by at least 3 weeks postnatal age. With this new protocol, it was decided to advise maternity service providers of this broader timeline of 36 to 72 h for routine collections as part of the updated collection guideline [17].

### 3.4. Adherence to New SP Protocol

In the year from May 2023 to April 2024 inclusive, 76,403 babies were screened. Of these, 668 babies (0.9%) had a recorded birth weight (BW) and/or gestational age (GA) that met the SP criteria (Table 3).

**Figure 2:**
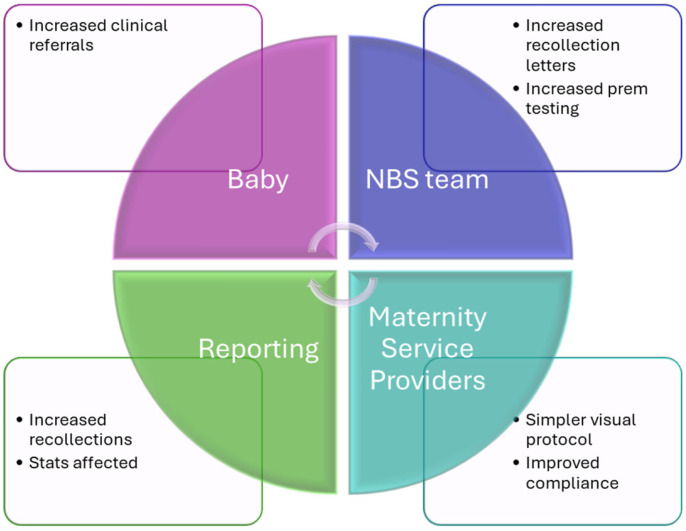
Organizational impact of SP protocol—protocol live from Monday 17 April 2023 [18]. We estimated about 160 babies in our jurisdiction received the additional collection over the 12-month follow-up audit period (May 2023 to April 2024).

This had a small organizational impact on the newborn screening program delivery for this small number (<1%) of babies. The organization impact tool allows an assessment of the impact of the change on other aspects of the process and includes here the impact on the baby, the changes to reporting processes and the workload for the various teams involved in NBS from collection through to the clinical follow-up. Details on organizational impact can be found at [18] (Figure 2).

## 4. Discussion

With the introduction of an increased number of conditions, and requests for a flow-diagram visualization of a combined protocol for preterm and sick neonates, we looked to undertake a full review of our current processes. This led to the successful introduction of our new sick–prem (SP) protocol, which is provided in Figure 1. The successful completion of our preterm and sick repeat collection audit has resulted in improved and clearer processes for the management of this cohort. This is supported by the increase in the previous 76% adherence to now achieving 95% collection of second samples in this cohort. It also demonstrates the importance of data-driven review for continuous improvement.

Screening for primary congenital hypothyroidism commenced in Victoria and all of Australasia in the 1970s [7,19,20,21]. At this time, the mortality for extremely preterm infants was high; however, with subsequent improvements in survival, consideration turned to improving morbidity and studies of thyroid function demonstrated differences in preterm infants compared to full-term neonates [10]. In preterm neonates, thyroxine levels are known to decrease in the first few weeks of life and the difference between preterm and full-term neonates persists until 30–49 days postnatally [22]. In a study of preterm neonates <30 weeks gestation conducted by our group a decade ago, we confirmed that serum-free thyroxine levels were as low as <2.6 pmol/L for the first 28 days with the nadir at 7 days. We also identified differences in the thyroid hormone concentration in the younger (23–26 weeks) compared to the older (27–29 weeks) preterm babies; i.e., fT4 levels were significantly lower in the younger group compared to the older preterm neonates [23]. Furthermore, the condition of transient hypothyroxinemia of prematurity (THOP) is now established and affects infants born at less than 30 weeks gestation. THOP is characterized by an initial rise in T4 at 24 h post-birth followed by a decline compared to cord blood levels with a nadir at 7 days of age, and TSH secretion at this time sees a return of T4 levels; however, in the sickest preterm neonates, this 24 h surge is often absent. An association also exists between THOP and adverse outcomes among the preterm neonates and this association is strongest for babies born at less than 28 weeks gestation [22]. We compared this combined evidence with the CLSI recommendations for timing of follow-up collection “at 28 days of age or discharge, whichever comes first” [1]. This CLSI advice was the basis of our timing for follow-up collections in our preterm algorithm given the research-based evidence found on the time the thyroid axis typically starts behaving as expected for a full-term neonate. Following the update of our protocol, the AAP guideline for CH screening was released and it is concordant with our decision of retesting babies who at birth were <1500 g and/or <32 weeks GA [2].

Very or extremely low birth weight has been used as the decision point for our program, but some other programs also include gestational age-related decision points. On reviewing the CLSI guideline cutoffs of 2000 g and 34 weeks for rescreening of thyroid panel and CAH, we noted these did not coincide with the definitions of the WHO [1,15,16]. Across Australasia, repeat screening is harmonized to babies with a birthweight of <1500 g, which is consistent with others [2,7,24]. With a view to staying harmonized with other Australasian NBS programs, and consistent with current neonatal definitions, we decided to stay with the <1500 g cut point for the routine repeat collection [15]. However, during the audit, we noted a few babies who had a GA recorded without a birthweight and given the recommendation in the CLSI guideline to include GA, we added a GA < 32 weeks for follow-up collection; i.e., the WHO cut point for very preterm [16]. The addition of GA only captured an extra 5% of babies and therefore the inclusion of this extra parameter does not significantly influence the number of repeat collections required. Overall, our decisions here are around harmonization as the evidence for appropriate cut points becomes a compromise between all the conditions screened and clinical risk.

The evidence around the decision and timing to recollect for transfusions was scant. In our jurisdiction, we had been performing a transfusion protocol for all babies that had had any form of transfusion. On reviewing the CLSI guideline, the focus was on red blood cell (RBC) transfusions and extracorporeal life support (ECLS), indicating that a RBC transfusion invalidates multiple tests for newborn screening [1]. Hence, using the advice in this guideline, we pulled back to only requesting transfusion repeat collections related to babies who had received RBC or ECLS. We then consulted with the HGSA NBS Committee on the rationale for repeat collections, especially timing, and we found our practice extended longer than in other jurisdictions, and while there was limited evidence provided for the timing of follow-up collections, we moved to harmonize with the other jurisdictions in Australasia and changed from 3 weeks to 1 week post-transfusion for a follow-up collection. We note, however, that further transfusion-related repeat collections may need to be considered in association with the proposed introduction of sickle cell screening [25].

The change to the timing of collection was implemented at the start of the COVID-19 pandemic in 2020 and formalized for all babies as part of this SP introduction [26]. This was carried out in the background using evidence from New Zealand and Western Australia. The aim of this change was to screen the babies before leaving the hospital. Overall, our data indicates that results in the window of 36–72 h are consistent with the traditional window of 48–72 h, and with the trend of earlier discharge from hospital this change has been seen as an advantage to the maternity service providers’ workflow. Notably, other programs in Australasia have a similar expanded window, but continue to state 48–72 h on their information [7]. Advertising this change did result in feedback related to the hospital’s ability to be agile with updating in-house documentation (which stated 48–72 h), but as this timeframe was only extended, the hospital’s documentation could be updated with the next review cycle.

There were a few limitations to this study and protocol review. The first one relates to the design of the original preterm baby second collection review, which looked at only the receipt of a second collection and not the actual timing of the second sample. This could mean that the overall adherence rate could be lower than the 76% of babies identified in this study. The second limitation relates to the unknown survival rate of this cohort, and this may have influenced the repeat collection numbers. Finally, while this review was performed in conjunction with the implementation of CAH screening, the information provided in the CLSI guideline recommended 4 weeks post-birth for a follow-up collection. However, it is well established that the fetal adrenal gland persists until at least the equivalent of term despite early delivery. Hence, 4 weeks for extreme preterm neonates will not approach this milestone of full-term equivalent at 4 weeks of age and the evidence of ontogeny suggests that a longer time period for follow-up may be warranted. To date, given the rate of classical CAH approximates 1:15,000 and all pre-terms make up 8.2% of births, we have not yet identified a preterm baby with CAH and such babies identified should inform future practice. In the absence of evidence, harmonization of practice is the best we can achieve to ensure all babies, irrespective of the jurisdiction, receive the same care.

Overall, the implementation of this preterm protocol has led to a significant change that has in turn impacted the organization of our NBS program. In the pre-analytical phase, the higher compliance with repeat collections indicates successful integration of the new protocol across maternity provider services in our jurisdiction. The higher workload involved for staff who perform the repeat tests is not new, however, rather more consistently recognized and completed compared to pre-2023. However, the NBS laboratory team now need to manage and follow up the maternity service providers that fail to send in a timely repeat collection, and this has added to the overall workload. Post-analytically, more babies are likely to be referred and so the clinical teams will potentially be assessing more babies, but the sample size is too small to adequately review the impact currently. The benefit to the baby in doing more good than harm is paramount for our NBS program and the design of the SP protocol process map allows for easy change as we continue to review our processes and impact into the future.

## 5. Conclusions

We recommend NBS laboratories audit preterm and sick neonate repeat collections to ensure appropriate follow-up of babies. This should be supported with a visual process map to aid education and compliance.

## Figures and Tables

**Table 1:** WHO definitions for low birthweight [15] and prematurity [16].

Birthweight (BW)	Preterm (PT) by Gestational Age (GA)
Low BW < 2500 g Very Low BW < 1500 g Extremely Low BW < 1000 g	Moderate or late PT 32–<37 weeks Very PT 28–<32 weeks Extremely PT < 28 weeks

**Table 2:** Comparison of time of repeat collection guideline per jurisdiction for preterm and sick neonates ^1^. Note: all jurisdictions recommended a pre-transfusion sample for affected babies.

Jurisdiction	Preterm—Second Sample, Adapted from [7]	Post-Transfusion Sample
NSW	<1500 g or <30/40 weeks repeat at one month	48 h + 2 weeks
QLD	<1500 g repeat screen 14 days <1000 g repeat screen again 28 days	48 h + 2 weeks
SA	<1500 g repeat at 10 days and again at 30 days (or at discharge)	1 week
VIC	1000–<1500 g repeat at 2 weeks <1000 g repeat at 3 weeks	48 h + 3 weeks
WA	≤1500 g repeat screen 14 days ≤1000 g repeat screen again 28 days	48 h
NZ	≤1500 g repeat screen 14 days ≤1000 g repeat screen again 28 days	1 week
CLSI 2009 [1] *	<34 weeks GA or <2000 g repeat screen for thyroid and CAH and other unresolved tests at 28 days of age	120 days *
AAP 2023 [2] **	<32 weeks GA or <1500 g repeat screen for thyroid at 2–4 weeks of age Plus, further follow-up if baby has not reached 36 weeks corrected GA at time of second sample	2–4 weeks of age

^1^ HGSA meeting discussion—v3 new protocol and comparison—meeting 2 March 2023. The five jurisdictions in Australia are New South Wales (NSW), Queensland (QLD), South Australia (SA), Victoria (VIC), and Western Australia (WA). There is only one screening service for New Zealand (NZ). * This recommendation considers an extended screening panel that includes hemoglobinopathies. ** Recommendations specific for thyroid screening, published post our audit.

**Table 3:** One-year follow-up audit of SP protocol (May 2023 to April 2024) showing the n = 668 babies who met the inclusion criteria of GA < 32 weeks and/or BW < 1500 g. Data are shown divided into specific subsets of the inclusion criteria to demonstrate the makeup of this cohort. Overall, 95% had a second sample collected.

SP Criteria	First Sample	Second Sample	Percentage of Second Samples
GA < 32 weeks	566	546	96%
BW < 1500 g	635	606	95%
Both BW and GA	523	507	97%
GA < 32 weeks and no BW	4	N/A	N/A
BW < 1500 g and no GA	333	N/A	N/A

## Data Availability

Please email the corresponding author if you would like to discuss the background data further.
